# Comparative metabolomics of scab-resistant and susceptible apple cell cultures in response to scab fungus elicitor treatment

**DOI:** 10.1038/s41598-018-36237-y

**Published:** 2018-12-14

**Authors:** Amol Sarkate, Shashank Sagar Saini, Deepa Teotia, Mariam Gaid, Javid Iqbal Mir, Partha Roy, Pawan Kumar Agrawal, Debabrata Sircar

**Affiliations:** 1 Plant Molecular Biology Group, Biotechnology Department, Indian Institute of Technology Roorkee, Roorkee, 247667 India; 2 Institute of Pharmaceutical Biology, Technische Universität Braunschweig, D-38106 Braunschweig, Germany; 3 Plant Biotechnology Department, Central Institute of Temperate Horticulture (ICAR-CITH) Srinagar, 190 005 J&K, India; 4 Molecular Endocrinology Laboratory, Biotechnology Department, Indian Institute of Technology Roorkee, Roorkee, 247667 India; 5 Krishi Anusandhan Bhawan-I, ICAR, New Delhi, India

## Abstract

Apple scab disease caused by the fungus *Venturia inaequalis* is a devastating disease that seriously affects quality and yield of apples. In order to understand the mechanisms involved in scab resistance, we performed gas chromatography-mass spectrometry based metabolomics analysis of the cell culture of scab resistant cultivar ‘Florina’ and scab susceptible cultivar ‘Vista Bella’ both prior -to and -following treatment with *V*. *inaequalis* elicitor (VIE). A total 21 metabolites were identified to be altered significantly in ‘Florina’ cell cultures upon VIE-treatment. Among 21 metabolites, formation of three new specialized metabolites aucuparin, noraucuparin and eriobofuran were observed only in resistant cultivar ‘Florina’ after the elicitor treatment. The score plots of principal component analysis (PCA) exhibited clear discrimination between untreated and VIE-treated samples. The alteration in metabolite levels correlated well with the changes in the transcript levels of selected secondary metabolite biosynthesis genes. Aucuparin, noraucuparin and eriobofuran isolated from the ‘Florina’ cultures showed significant inhibitory effect on the conidial germination of *V*. *inaequalis*. The results expand our understanding of the metabolic basis of scab-resistance in apple and therefore are of interest in apple breeding programs to fortify scab resistance potential of commercially grown apple cultivars.

## Introduction

Apple (*Malus domestica*) is the main deciduous fruit crop of the temperate region of the world^1^. Apple fruits are rich in antioxidants and health protective polyphenols^2,3^. The major categories of polyphenols detected in apple are catechins, epicatechin, proanthocyanidins, flavonols, dihydrochalcones, hydroxycinnamates and anthocyanins^4–7^. In addition, apples are also rich source of many bioactive triterpenes^8^. Regular consumption of apples is known to be associated with the reduced risk of heart disease and cancer^9^. Apples and apple-derived products possess several health beneficial activities, such as anticancer, anti-diabetic, and cholesterol-lowering properties^10^.

The apple scab disease is caused by the ascomycetous fungus *V*. *inaequalis*. Scab is one of the most devastating diseases of apple in terms of economic damage^11,12^. The pathogenic phase of *V*. *inaequalis* infection initiates with the germination of ascospores (sexual spores), which serve as the primary source of infection on fallen leaves. The conidiospores (asexual spores) serve as a secondary infection source. In a single apple-growing season, multiple cycles of conidiospore production and secondary infections occurs^13^. The control of the disease relies mainly on multiple fungicides spray^14^, which exhibit toxic effect to human health and may even contaminate the environment. Moreover, extended use of scab controlling fungicides may result in selection pressure on the *V*. *inaequalis*, thereby inducing emergence of new resistant races^15^. Apple plants have natural defense systems against a wide range of pathogens. The genus *Malus* has coevolved with the scab fungus *V*. *inaequalis*, and many apple accessions harbor scab-resistance genes that are currently used for introgression from wild relatives to commercial cultivars through breeding programs^12,16^. In recent past, a number of scab-resistance genes (*Rvi1* – *Rvi17*) have been isolated and mapped from various scab-resistant cultivars across the globe^12^. Notably, until now *Rvi15* is the only gene available to provide complete resistance against all the available races of *V*. *inaequalis*^17,18^. Apple breeding programs usually take longer time and generally involve a series of back crossings between resistant and susceptible cultivars to recover near-isogenic lines expressing the desired commercial trait. Furthermore, self-incompatibility in apples make breeding process even more problematic as the elite varieties sometimes might not be recovered by crossing. As a result, strategies able to directly transfer traits to elite cultivated varieties through cis-genic approach would accelerate the breeding process^19^.

Several apple cultivars such as ‘Liberty’, ‘Florina’, ‘Prima’, ‘Sir Prize’ are resistant to scab infections. The disease resistance mechanism depends on the nature of genetic interactions between *V*. *inaequalis* and *Malus* species in accordance to the gene-for-gene concept^20^. To date, more than 17 ‘*R’* genes have been indentified from different *Malus* species providing varying levels of resistance against *V*. *inaequalis*^11,12^. The early stages of scab infection beginning with spore germination, appressoria formation, cuticular penetration, and fungal hyphae development are similar in both resistant and susceptible apple cultivars^21^. Subsequently, resistant cultivars show hypersensitive response (HR) whereas susceptible cultivars exhibit sporulating lesions and disease symptoms depending on the resistance genes, pathogen race, and ontogenic resistance^22^. The HR in apples are exhibited by several previously characterized ‘*R’* genes such as *Rvi4*, *Rvi5*, *Rvi7*, *Rvi10*, *Rvi15* and *Rvi16*^18^ that produces typical “pin-point pits”. These pin-point-pits are formed as a result of rapid death and subsequent collapse of cells immediately surrounding the germinating spore penetration site^23^. The HR prevents colonization of fungus through programmed cell death and may involve cell to cell signaling mediated by formation of reactive oxygen species and defense responsive metabolites such as phloretin, biphenyls, dibenzofurans and phenolics^24–26^. Phenolic metabolites produced in response to scab infections in apple are known to inhibit growth of *V*. *inaequalis*, suggesting their involvement in scab resistance^11^. Suppression of phenylalanine ammonia lyase, the key enzyme in phenolic biosynthesis, in the scab resistant apple cultivar ‘Sir Prize’ turned it to scab susceptible^27^. The breakdown of phloridzin into phloretin by *V*. *inaequalis* is known to be associated with hindering fungal growth^28^. Earlier, it was reported that the treatment of *V*. *inaequalis* elicitor induces synthesis of salicylic acid in the cell suspensions of apple cultivar ‘Florina’^29^. The elicitor-induced formation of a novel phytoalexin malusfuran was reported from the cell suspensions of scab resistant cultivar ‘Liberty’^28^. Malusfuran was found to suppress the rate of *V*. *inaequalis* conidia germination. However, systematic understanding of the defense-responsive metabolites underlying scab-resistance in apples is still elusive. It was also reported that cell wall surrounding the scab infection site undergoes rapid lignifications to reduce the risk of secondary infections in many scab resistant cultivars^30^. In addition to HR, the other resistance phenotypes such as chlorotic flecking^31^ and necrotic flecking^32^ have been observed in the selected cultivars. Previously, it was reported that pathogenicity related proteins like *β*-1,3-glucanase, chitinase, and thaumatin-like protein were constitutively expressed in the apoplast of the resistant apple cultivar ‘Remo’ and were induced in susceptible cultivar ‘Elstar’ after *V*. *inaequalis* infection^33^. The transgenic apple lines of scab susceptible cultivar ‘McIntosh’ expressing either the endo (*ech42*) or the exo (*nag70*) chitinase gene of *Trichoderma harzianum* are shown to be scab tolerant^34^. Similarly cis-genic apple lines of the cultivars ‘Brookfield Baigent’, ‘Mitchgla’, ‘Novajo’, and ‘Pinova’ harboring *Rvi6* gene showed enhanced scab resistance^19^.

Metabolomics is widely used to investigate the tolerance of plants to biotic stresses^35^. After pathogen infection, the plant starts synthesizing an array of defense-responsive metabolites, both primary and secondary. Alteration in the metabolic profiles of a plant in response to pathogen infection can be analyzed with metabolomics approach. This technology provides the opportunity to evaluate pathogen-induced local and systemic alterations in plant metabolite patterns without any prior assumptions. Measuring the level of metabolites prior and after pathogen infection may provide an exact status of the physiological condition of the plant tissue under consideration. The gas chromatography-mass spectrometry (GC-MS) is routinely used in plant metabolomics^36^, especially for facilitating the identification and quantification of the primary metabolites such as amino acids, sugars, organic acids^37^ and an wide array of secondary metabolites like phenolics, flavonoids, terpenes and specialized phytoalexins. Most metabolomics based studies on apples published so far were targeted analyses focusing on specific metabolites and limited to fruit quality^38–40^. Recently NMR based metabolomics were performed to identify new scab-preventive metabolites from scab resistant apples^41^. However, untargeted metabolite analysis provides a more comprehensive view on the differential accumulation of metabolites upon pathogen infection^32^.

We hypothesized that scab resistant and scab susceptible cultivars of apple would respond differentially towards scab infection in terms of metabolic re-programming and for that a non-targeted GC-MS based metabolomics approach can be used. To investigate this hypothesis, we employed non-targeted comparative GC-MS to identify scab-responsive metabolites from the VIE*-*treated cell suspension culture of scab resistant and susceptible apple cultivars namely ‘Florina’ and ‘Vista Bella’ respectively. Comparative metabolomics resulted in a profile of total 60 metabolites from the ‘Florina’ cell cultures after VIE treatment, out of which 21 metabolites showed strict differential accumulation in the ‘Florina’. Based on these results, selected secondary metabolites were chosen for analyzing their inhibitory effect on the *V*. *inaequalis* conidial germination. Information derived from this study will provide new insights on scab-resistant mechanisms in apple in terms of metabolites.

## Results

### Comparative metabolomics of VIE-treated apple cell cultures

Cell cultures of scab resistant (SR) apple cultivar ‘Florina’ and scab susceptible (SS) cultivar ‘Vista Bella’ was used for comparative metabolomics. In order to understand metabolic re-programming following VIE-treatment, metabolomics was performed on samples prepared from treated SR and SS cell cultures at 6, 12, 24, 36, 48 and 72 hours post elicitation (hpe) and control (SR0 and SS0) untreated cultures. After double derivatization, metabolites were profiled by GC-MS metabolomics analyses, comparing the levels of each metabolite at defined time points to the equivalent un-treated controls. Representative GC-MS chromatograms of ‘Vista Bella’ and ‘Florina’ cell cultures are shown in Supplementary Fig. S1. A total of 60 low-molecular weight metabolites were detected in the ‘Florina’ cell cultures, whereas 55 metabolites were detected in the ‘Vista Bella’ cultures as shown in Supplementary Table S1.

In the next step, we identified VIE-responsive metabolites that showed significant (*p* ≤ 0.05) differential concentrations between SR and SR0 and between SS and SS0. A total of 31 and 10 differentially accumulating metabolites were identified in ‘Florina’ and ‘Vista Bella’ cell cultures, respectively, as shown in Table 1. Statistically significant differentially accumulating metabolites detected from ‘Florina’ were further analyzed by employing Bonferroni correction (*p* ≤ 0.05) and their adjusted *p*-values were shown in Supplementary Table S2. Detected metabolites were further classified into specific metabolite classes such as amino acids, sugar alcohols, sugars, organic acids, vitamins, phenolics and secondary metabolites, on the basis of their chemical nature (Table 2). Metabolite feature areas were normalized using the area of internal standard before performing comparative metabolomics.

**Table 1:** List of differentially accumulating metabolites detected from VIE-treated cell cultures of ‘Florina’ and ‘Vista Bella’.

S.No.	Differentially accumulating metabolite	TMS Derivate	KEGG/PubChem ID	Retention Time	Qualification Ions [*m/z*]	Significance of differential accumulation	
						‘Florina’	‘Vista Bella’
1	Aspartic acid	3 TMS	529617	13.52	349, 218	S	S
2	Ascorbic acid^a^	4 TMS	C00072	18.26	464, 449	S	NS
3	Aucuparin^a^	1 TMS	CID 442508	24.08	302, 287	S	ND
4	Benzoic acid^a^	1 TMS	C00180	13.6	194, 179	S	NS
5	Caffeic acid	3 TMS	C01197	22.86	396, 381	S	S
6	Catechin^a^	5 TMS	C00199	26.25	649, 576	S	ND
7	Chlorogenic acid	6 TMS	C00852	21.24	786, 712	S	S
8	Citric acid^a^	4 TMS	C00158	16.53	465, 437	S	NS
9	p-Coumaric acid^a^	2 TMS	C00811	22.2	308, 293	S	NS
10	trans-Cinnamic acid^a^	1 TMS	C00423	17.81	220, 205	S	NS
11	Eriobofuran^a^	1 TMS	CID 178939	25.19	316, 288	S	ND
12	Ferulic acid	2 TMS	C01494	23.6	338, 323	S	S
13	Fructose^a^	5 TMS	C00095	19.61	569, 307	S	NS
14	Fumaric acid	2 TMS	C00122	14.85	260, 245	S	S
15	Glucose	6 TMS	C00031	19.86	540, 525	S	S
16	Malic acid^a^	3 TMS	C00149	16.69	350, 335	S	NS
17	Malonic acid	3 TMS	C00383	15.51	305, 231	S	S
18	Mannitol^a^	6 TMS	C00392	24.85	421, 319	S	NS
19	Mannose^a^	5 TMS	C00159	18.81	435, 393	S	NS
20	Noraucuparin^a^	2 TMS	CID44605718	23.8	330, 313	S	ND
21	Phenylalanine^a^	2 TMS	C00079	16.09	294, 266	S	NS
22	Proline	2 TMS	C00148	11.85	259, 216	S	S
23	Protocatechuic acid^a^	3 TMS	C00230	20.82	370, 355	S	NS
24	Pyruvic acid	1 TMS	C00022	10.78	145, 116	S	S
25	3-phosphoglyceric acid^a^	3 TMS	C00197	14.58	402, 337	S	NS
26	Salicylic acid^a^	2 TMS	C00805	17.2	281, 267	S	ND
27	Serine^a^	3 TMS	C00065	12.75	306, 278	S	NS
28	D-Sorbitol^a^	6 TMS	C00794	19.48	217, 147	S	NS
29	Sucrose	8 TMS	C00089	26.79	437, 361	S	S
30	Succinic acid^a^	2 TMS	C00148	12.03	262, 247	S	NS
31	Tryptophan^a^	3 TMS	C00078	20.29	405, 291	S	NS

S: significant (*p* < 0.05); NS: non-significant (*p* > 0.05); ND: not detected. ^a^denotes metabolites showing differential accumulation only in ‘Florina’.

**Table 2:** Total number of identified metabolites within each metabolite class.

Class of metabolites	Number of identified metabolites in ‘Florina’	Number of identified metabolites in ‘Vista Bella’
Amino acids	7	7
Fatty acids	3	3
Organic acids	14	14
Sugar alcohol	4	4
Sugars	11	11
Vitamins	1	1
Phenolics	10	8
Biphenyls-dibenzofuran phytoalexins	3	0
Other metabolites	7	7
**Total**	60	55

**Figure 1:**
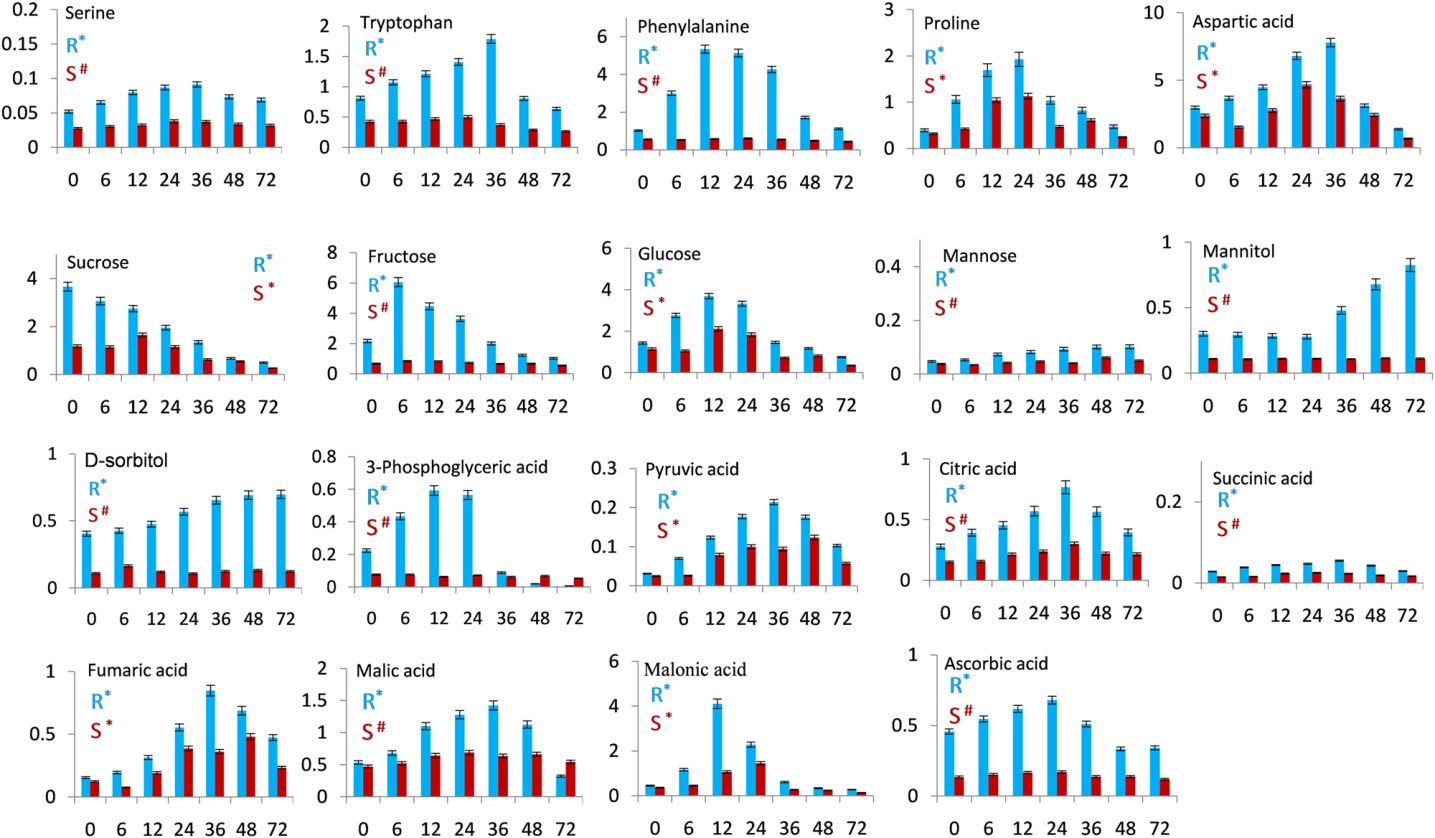
Differentially accumulating primary metabolites in the VIE-treated cell cultures of apple cultivar ‘Florina’ [blue; resistant cultivar (R)] and ‘Vista Bella’ [red, susceptible cultivar (S)]. Data are presented as mean values and error bars represent the standard deviation. The x-axis represents post-elicitation time points (h). The y-axis gives the normalized relative metabolite abundance in terms of the area of internal standard phenylphenol. The ANOVA was performed to assess the statistical significance of differences between samples at different time points (*p* < 0.05). *Denotes significant difference (*p* < 0.05); ^#^Denotes non-significant difference (*p* > 0.05).

**Figure 2:**
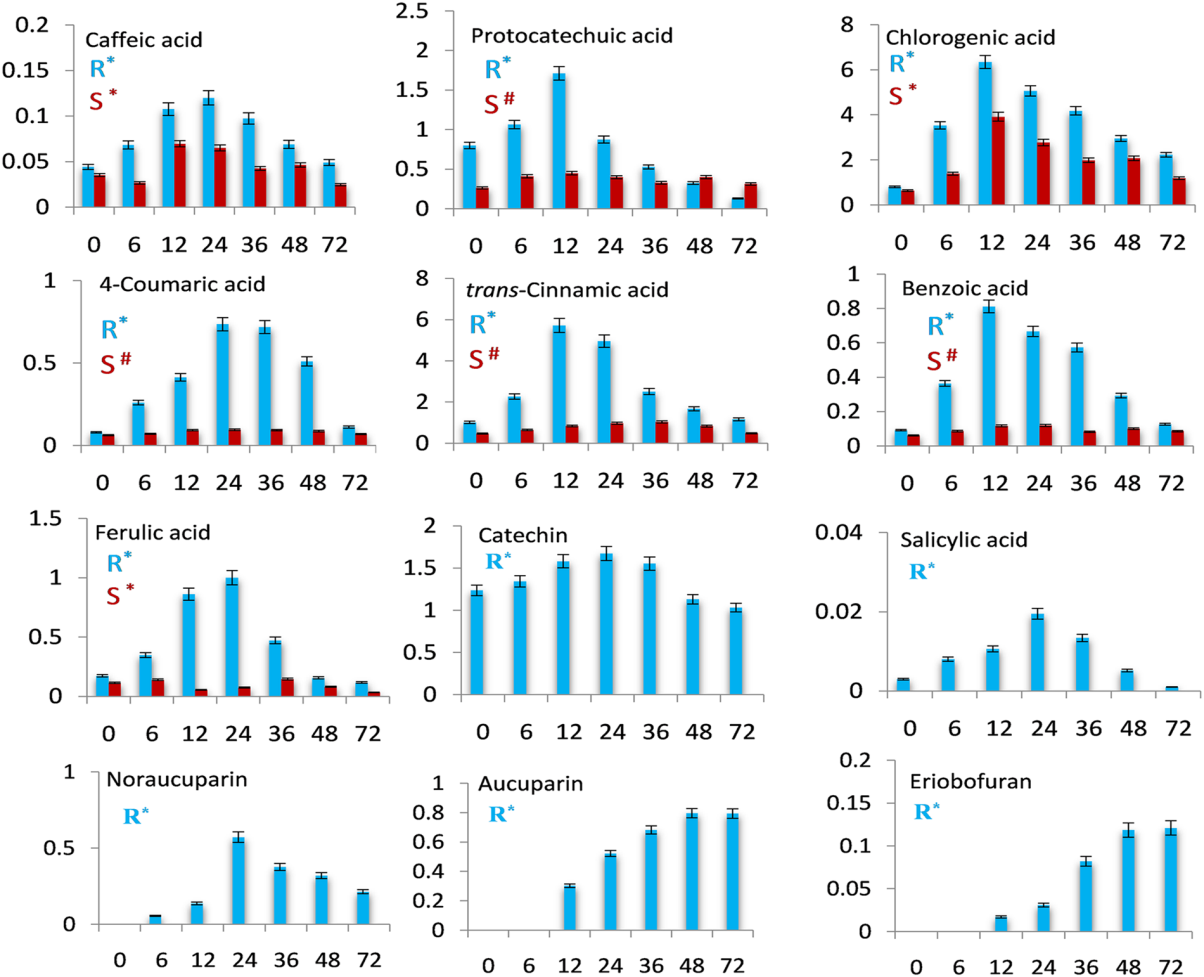
Differentially accumulating secondary metabolites detected from the VIE-treated cell cultures of ‘Florina’ (blue) and ‘Vista Bella’ (red). The x-axis represents post-elicitation time points (h). The y-axis gives the normalized relative metabolite abundance in terms of the area of internal standard phenylphenol. The ANOVA was performed to assess the statistical significance of differences between samples at different time points (*p* < 0.05). *Denotes significant difference (*p* < 0.05); ^#^Denotes non-significant difference (*p* > 0.05).

As shown in Fig. 1 and Fig. 2, all of the differentially accumulating metabolites did not show distinct accumulation patterns in both SR and SS cell cultures during the time course studied. Interestingly, metabolites belonging to the same biosynthetic pathway often showed distinct accumulation patterns during different post elicitation time points. After VIE-treatment, most of the differential metabolites started enhanced accumulation. At 12 or 36 hpe most of the differential metabolites showed maximum accumulation in SR cultures, including the amino acids, organic acids and phenolics. Following the progression of elicitation, metabolite level first dramatically increased to attain a peak and then decreased thereafter at 72 hpe. Notably, a large number of differentially accumulating metabolites in ‘Florina’ cultures that showed elevated levels, have been identified in more than one time point following elicitation, suggesting activation of the metabolic pathways leading to the biosynthesis of these particular metabolites.

### VIE-treatment changes the primary and secondary metabolite signature in ‘Florina’ and ‘Vista Bella’ cultures

VIE-treatment appears to trigger massive metabolic re-programming in ‘Florina’ cell cultures both at the level of primary and secondary metabolites (Fig. 1 and Fig. 2). In comparison, the numbers of metabolites showing a significant differential accumulation in VIE-treated SR compared to SR0 cultures were much lower in VIE-treated SS versus SS0 cultures. Among the 31 differentially accumulating metabolites detected from ‘Florina’, 30 metabolites were up-regulated and one metabolite was down-regulated as shown in Fig. 1. On the contrary only nine metabolites were significantly up- and one metabolite was down-regulated in ‘Vista Bella’ cultures after VIE-treatment (Fig. 1 and Fig. 2). Among the amino acids detected in ‘Florina’, the levels of serine, tryptophan, phenylalanine, proline and aspartic acid were significantly up-regulated after VIE-treatment. Similarly, VIE-treatment triggers up-regulation of glucose, fructose and mannose contents; whereas sucrose content was down-regulated. Among the sugar alcohols, mannitol and sorbitol contents were significantly up-regulated. Pyruvic-, 3-phosphoglyceric-, citric-, succinic-, fumaric-, malic-, and malonic acid levels were up-regulated. Among the vitamins, only ascorbic acid was detected from ‘Florina’ cell cultures, whose level was significantly up-regulated after elicitor treatment. Phenolics and specialized biphenyl and dibenzofuran phytoalexins were the most abundant secondary metabolites detected in the VIE-treated ‘Florina’ cell cultures. Plant phenolics are known to play crucial role in pathogen defense^42^. Nine phenolics and three inducible phytoalexins (two biphenyl phytoalexins: noraucuparin and aucuparin; one dibenzofuran phytoalexin: eriobofuran) were detected. All these phenolic metabolites showed differential accumulation after the VIE-treatment. The content of these phenolics were first up-regulated after the VIE-treatment and thereafter declined to the basal level (Fig. 2). Interestingly, in our study, biphenyl and dibenzofuran phytoalexins were not detected from the untreated control cells and formed in ‘Florina’ cultures only after the VIE-treatment. On the contrary, in scab susceptible ‘Vista Bella’ cell cultures, only differential accumulation of caffeic-, chlorogenic- and ferulic acids were observed (Fig. 2). Catechin, salicylic acid, noraucuparin, aucuparin and eriobofuran were not detected from ‘Vista Bella’ cultures.

### Principal component analysis (PCA) reveals metabolic alterations in VIE-treated ‘Florina’ cell cultures

**Figure 3:**
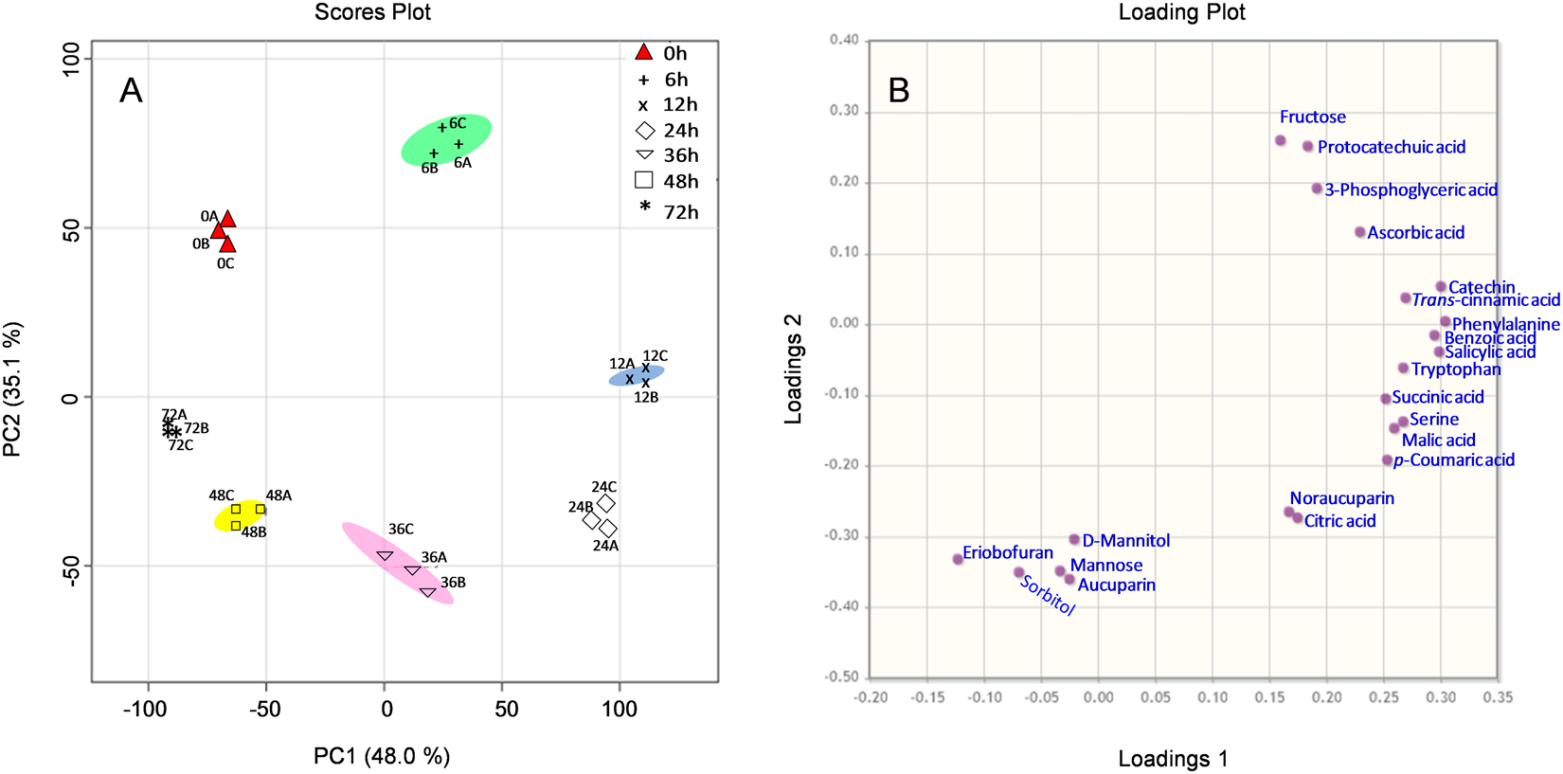
Scores plot (**A**) and loading plots (**B**) of principal components (PC1) and (PC2) from the PCA results obtained from 21 differentially accumulating metabolites detected from the VIE-treated cell cultures of scab resistant cultivar ‘Florina’.

To assess the data reproducibility in the different biological replicates of the metabolites measurements, GC-MS data were subjected to principal component analysis (PCA). For PCA, only those 21 metabolites were included (^a^ marked in the Table 1) which showed differential accumulation only in the scab resistant cultivar ‘Florina’ but not in the scab susceptible cultivar ‘Vista Bella’. PCA helps to reduce the dimensionality of complex data sets, which facilitate better visualization of the inherent patterns in the data. PCA analyses uses, linear orthogonal transformation of the original data variables to generate a new set of uncorrelated variables known as principal components (PCs)^43^. Results indicated that the biological replicates for each group [control (0 h) and treated (6 h, 12 h, 24 h, 36 h, 48 h and 72 h)] were always clustered together (Fig. 3A), indicating on one hand the high data reproducibility at the different treatment time and on the other hand the significance of their effects on the metabolite level alteration. As shown in Fig. 3A, the first and second PCs of the analyzed PCA score plot represented 48.0% (PC1) and 35.1% (PC 2) of the total variance of the samples. Furthermore, a PCA loading plot (Fig. 3B) was constructed to show the abundant variable (metabolites) contributing to the PCA results. Our results suggest that these differentially accumulating metabolites are the probable reasons contributing to the resistance responses of ‘Florina’ against the VIE-treatment.

### Hierarchical clustering analysis of the metabolite profiles

**Figure 4:**
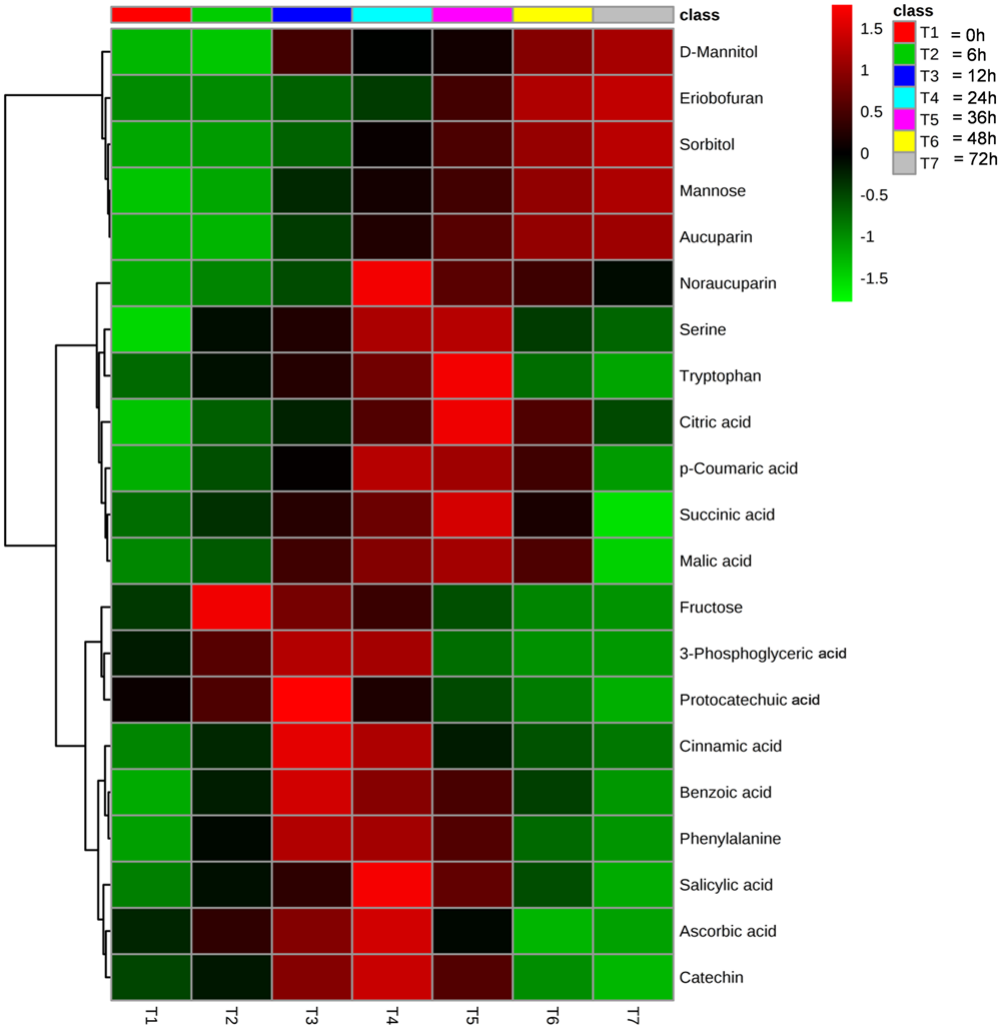
Heatmap analyses of 21 differentially accumulating metabolites from VIE-treated cell culture of ‘Florina’. Similarity assessment for clustering was done on the basis of Euclidean distance coefficient. Rows and columns represent individual metabolites and average samples, respectively.

In order to search for any probable discrepancies in the metabolite profiles of all the seven (0–72 h) sample groups, the 21 differentially accumulating metabolites used for the PCA analyses were organized and visualized by heatmap analysis tool of MetaboAnalyst 4.0 (Fig. 4)^44^. The Heatmap offered excellent separation of the metabolite trend between the non-treated (0 h) and VIE-treated (6–72 h) samples.

### Changes in metabolic pathways: metabolic pathway network

**Figure 5:**
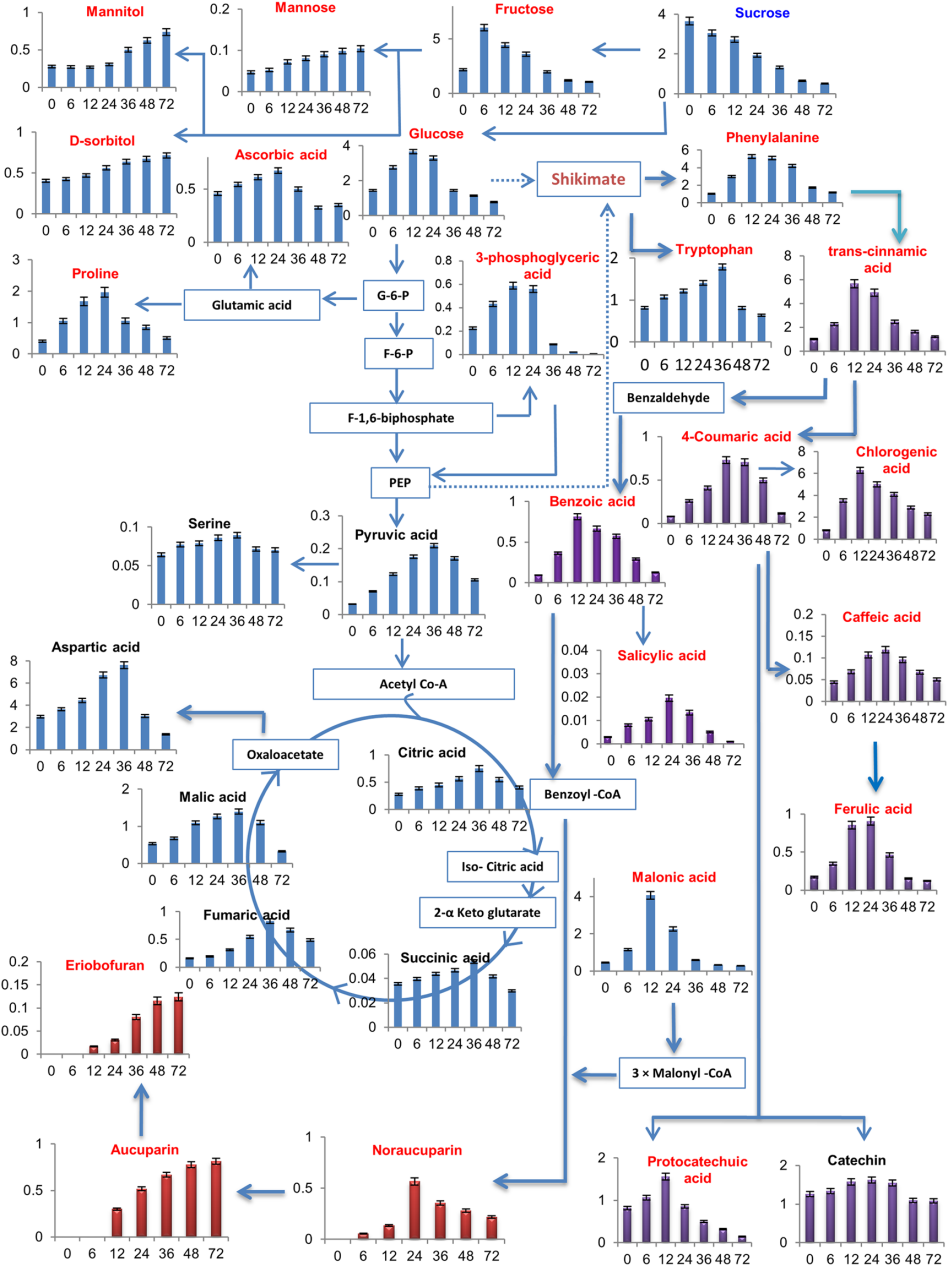
Schematic diagram of the metabolic pathway and relative levels of major metabolites detected in the VIE-treated cell cultures of ‘Florina’. The significantly up- and down-regulated (*p < *0.05) metabolites after VIE-treatment were indicated in red and blue, respectively.

A simplified metabolic pathway network was reconstructed using key metabolic pathways, such as the shikimic acid pathway, the phenylpropanoid pathway, the glycolytic pathway, the pentose phosphate pathway, the biphenyl biosynthetic pathway, the flavonoids biosynthesis pathway, the tricarboxylic acid (TCA) pathway, and the amino acid biosynthetic pathway to show the regulated pattern diversity of each detected differentially accumulating metabolites from ‘Florina’ with respect to their proportional incorporation into key metabolic pathways. As shown in Fig. 5, sucrose as the precursor for glucose and fructose was higher in non-elicited cells than that of VIE-treated cells. However, sucrose hydrolysis product, the glucose and the fructose were higher in the VIE-treated cells, suggesting that more active sucrose catabolism occurred in the VIE-treated cells. The level of mannose, sorbitol and mannitol were higher in the VIE-treated cells, suggesting that fructose is metabolized more towards these metabolites rather than re-entering into glycolytic pathway. The level of identified TCA cycle metabolites such as citric, succinic, fumaric and malic acids were up-accumulated in the VIE-treated cells suggesting higher turn-over number of TCA cycle. The metabolites derived from shikimate pathway showed an absolutely distinct accumulation pattern. The level of all the detected shikimate-derived metabolites such as caffeic acid, protocatechuic acid, catechin, chlorogenic acid, benzoic acid, ferulic acid, p-coumaric acid, *trans*-cinnamic acid and salicylic acid were higher in the VIE-treated cells. Aucuparin, noraucuparin and eriobofuran were synthesized only after the VIE-treatment.

### Gene expression analyses

**Figure 6:**
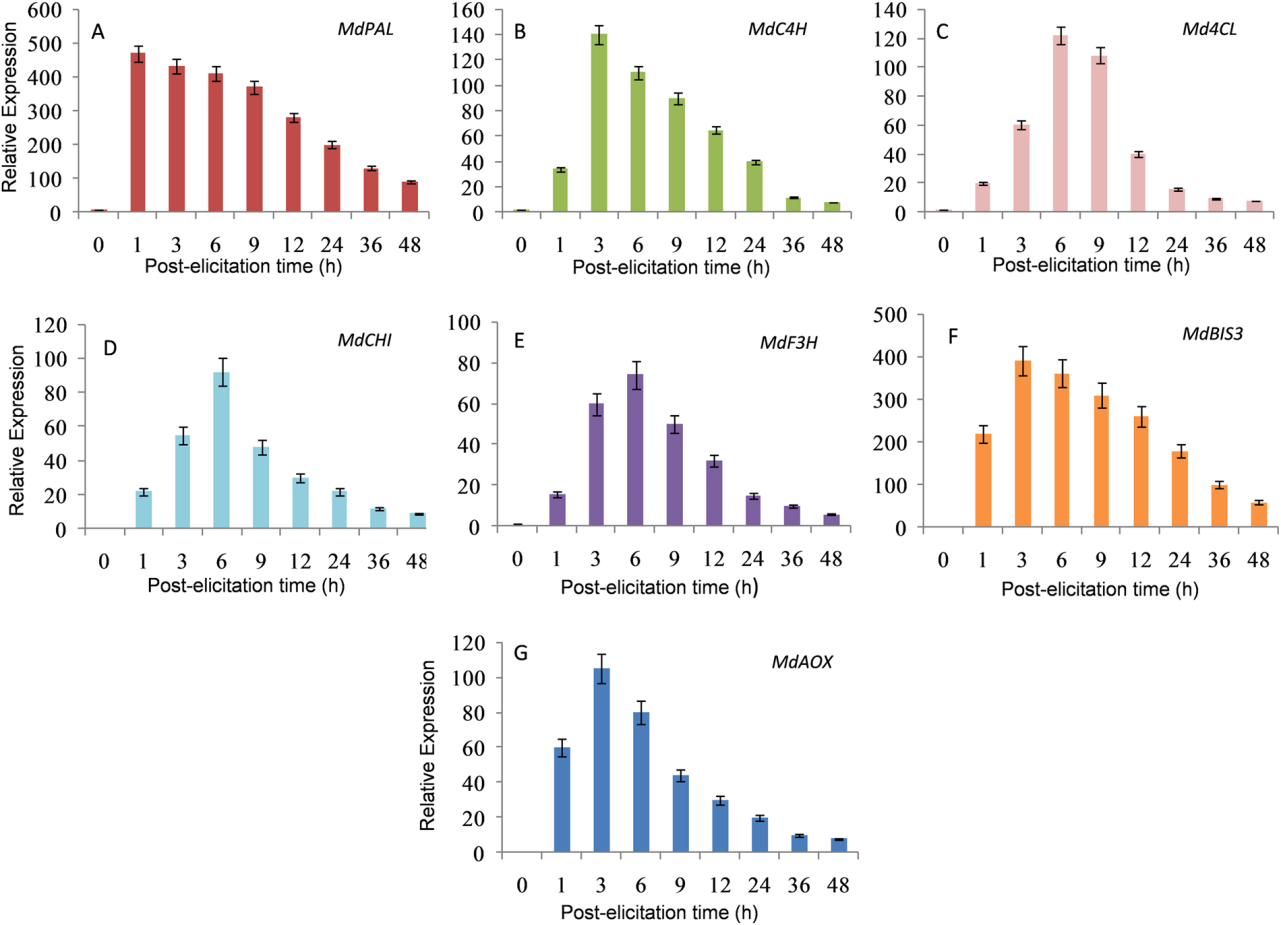
Changes in the gene expression level of *MdPAL*, *MdC4H*, *Md4CL*, *MdCHI*, *MdF3H*, *MdBIS3*, and *MdAOX* in the VIE-treated cell cultures of ‘Florina’. The relative transcript levels were determined by real-time-PCR. Results are means ± SD (*n* = 3).

The expression levels of selected genes coding for enzymes involved in phenylpropanoid, flavonoids and biphenyl biosynthesis were studied further as the contents of phenolics, flavonoids and biphenyls were found to be significantly elevated in the VIE-treated ‘Florina’ cell cultures. The expression levels of seven genes after VIE-treatment were investigated by quantitative real-time PCR, and the results are shown in Fig. 6. The expression of *phenylalanine ammonia lyase* (*MdPAL*) gene was markedly increased after the VIE-treatment. The expression level of *cinnamate-4-hydroxylase* (*MdC4H*), *4-coumarate:CoA ligase* (*Md4CL*), *chalcone flavanone isomerase* (*MdCHI*), *flavanone 3-hydroxylase* (*MdF3H*), *biphenyl synthase 3* (*MdBIS3*), and *alternative oxidase* (*MdAOX*) were also significantly up-regulated after VIE-treatment demonstrating that phenylpropanoid, flavonoid and biphenyl biosynthesis were triggered by the VIE-treatment. These gene expression data were well correlated with the enhanced accumulation of phenylpropanoids such as cinnamic acid, flavonoids and biphenyls in the elicited cell cultures of ‘Florina’.

### Inhibition of conidial germination of *V*. *inaequalis* with apple cell culture metabolites

The incubation of *V*. *inaequalis* conidial suspension with combinations of aucuparin, noraucuparin and eriobofuran, three induced metabolites detected from the VIE-treated ‘Florina’ cell culture resulted into 74% inhibition of conidial germination in contrast to the untreated control (Supplementary Fig. S2). Treatment of conidial suspension with noraucuparin (5 µM) alone resulted into 28% inhibition of conidial germination. Individual treatment with aucuparin (5 µM) and eriobofuran (5 µM) showed 46% and 34% inhibition in conidial germination, respectively. Combinations of aucuparin (5 µM) and noraucuparin (5 µM) resulted into relatively higher inhibition of conidial germination. For conidia germination assay, pH range of 5 to 5.6 was found to be optimum. These results clearly showed synergistic effect aucuparin, noraucuparin and eriobofuran on inhibition of *V*. *inaequalis* conidial germination. However the underlying mechanisms of actions for this inhibitory effect remain elusive.

## Discussion

The present GC-MS-based metabolomics analysis was performed to identify signature metabolites associated with apple resistance to scab fungus *V*. *inaequalis*. This study is the first report on the systemic identification of metabolic changes in response to VIE-treatment in the cell cultures of scab resistant cultivar ‘Florina’ and scab susceptible cultivar ‘Vista Bella’, before and after treatment. In this study we have used apple cell culture as model system because cell cultures offer an uniform plant material for studying metabolic changes and typically plant cell cultures exhibit relatively less metabolic complexity due to lack of pigments and lignified tissues. Earlier plant cell cultures have been successfully used for analyzing plant-microbe interactions in apple and other plant systems^29,45,46^. The metabolomics data were then correlated with the expression patterns of genes linked to selected secondary metabolite biosynthesis to obtain a comprehensive insight of the changes occurring after VIE treatment.

From the metabolic point of view, VIE-treatment appears to cause massive metabolic re-programming in ‘Florina’ both at the level of primary and secondary metabolisms. In this study we have detected only 60 metabolites from the VIE-treated cell cultures of ‘Florina’ which is relatively less as compared to the previously reported metabolites from apple^38^. This is possibly explained by the fact that cell culture system used in this study is devoid of complex metabolites such as chlorophyll, pigments and lignified tissues, and thereby represents a metabolically simple system as compared to intact apple plants and fruits.

The changes in the metabolites concentration suggested that six biochemical pathways were mainly altered by VIE-treatment. Considering the limited number of metabolites reported in this study, the most altered pathways were amino acid, sugar, sugar alcohols, organic acids, vitamin, phenolics and biphenyl-dibenzofuran phytoalexin biosynthesis. Earlier studies have shown that up-regulation of primary metabolites, mainly in the level of amino acids, sugar and sugar alcohols occur during plant-pathogen interactions^47^. Notably, VIE-treated ‘Florina’ cultures showed differential accumulation of selected organic acids. The elevated level of organic acids might be associated with the higher turnover number of glycolysis and TCA cycles, which generates more abundant biosynthetic intermediates. High organic acid concentrations are known to facilitate better ion absorption and thereby known to be associated with enhanced disease resistance^48^. Likewise, ascorbic acid is known to be associated with the superior defense responses in many plant species^49^.

Among secondary metabolites, phenolic and flavonoid contents were significantly up-regulated in the VIE-treated ‘Florina’ cell cultures as compared to ‘Vista Bella’ cultures. Similar array of polyphenols were previously detected in ‘Florina’ leaves^50^. However, quercitin and avicularin as detected earlier from ‘Florina’ leaves were not detected from VIE-treated ‘Florina’ cell cultures. Phenolic acids are known to be deposited along the cell wall to provide first line of defense against infection^51^. Previously it was reported that differential accumulations of flavonols (catechins and proanthocyanidins) were key factors in providing scab resistance in the apple cultivars^27,52,53^. In our study, high basal level of catechin content was also observed in the ‘Florina’ cell culture. Earlier finding showed that scab-resistant apple cultivars are particularly rich in the content of caffeic-, chlorogenic-, and ferulic acids and their concentration rapidly increases after the scab-infection as compared to susceptible cultivars^54^. It is known that benzoic acid serves as the precursor for biphenyl and dibenzofuran phytoalexins biosynthesis in apple^7^. Therefore, enhanced biosynthesis of benzoic acid in the VIE-treated ‘Florina’ cultures could be linked with bipehnyl and dibenzofuran phytoalexins formation in the ‘Florina’. It has already been reported that *trans*-cinnamic acid serves as the precursor of many defense metabolites, such as phenylpropanoids, benzoates, lignans, and flavonoids^55^. It was worth speculating that after the VIE-treatment; first *trans*-cinnamic acid level is up-regulated, which in turn get metabolized to biphenyl and dibenzofuran phytoalexins through intermediate formation of benzoic acid. Moreover, considerable up-regulation of salicylic acid (SA) biosynthesis was also observed in the VIE-treated ‘Florina’ cell culture. The salicylic acid can induce local resistance in the form of hypersensitive reaction or long distance systemic acquired resistance (SAR) by triggering the production of pathogenesis-related proteins^56^. The crucial role of SA as signaling molecule in plant defense response has already been reported earlier^57^. VIE-treatment to ‘Florina’ cell culture led to the production of biphenyl and dibenzofuran phytoalexins which are known to inhibit the growth of *V*. *inaequalis*^7^. Interestingly, in our study, biphenyl and dibenzofuran phytoalexins were absent from the untreated controls cells and from the scab susceptible cultivar ‘Vista Bella’. Notably, malusfuran, a glucosylated dibenzofuran phytoalexin detected earlier from the elicited cell culture of resistant apple cultivar ‘Liberty’, has not been detected in this study. On the contrary, eriobofuran (free dibenzofuran) has been detected from the VIE-treated ‘Florina’ cell cultures. The glucosylated dibenzofurans are possibly broken-down to provide free dibenzofuran such as eriobofuran upon pathogen infection. An identical breakdown of phloridzin into phloretin was observed in several scab resistant apple cultivars^58^.

During the present study, PCA was performed to evaluate the dynamics of the metabolic re-programming following VIE treatment (Fig. 3A). The metabolite profiles at 6, 12, 24, 36, 48 and 72 h reflected VIE-induced enhanced accumulation of metabolites as compared to non-treated 0 h control. Only, three metabolites, noraucuparin, aucuparin and eriobofuran were newly formed after VIE-treatment. The 21 detected metabolites that showed significant differential accumulation in SR cultivar ‘Florina’ indicates a strong defense response of SR cultivar to VIE-treatment. In addition, heatmap analyses (Fig. 4) demonstrated that the VIE-treated cells were the major sources of variance in the metabolic pool, which indicated significant metabolic re-programming by the VIE-treatment.

We additionally examined the qRT PCR-based selected gene expressions of ‘Florina’ cultures before and after the VIE-treatment. The expression levels of all the seven genes studied were up-regulated after the VIE-treatment, thereby indicating up-regulation of phenylpropanoid, flavonoids and biphenyl biosyntheses after VIE-treatment. Enhanced expressions of *PAL*, *C4H* and *4CL* genes are known to be associated with enhanced phenylpropanoid biosynthesis^59,60^_._ Likewise, high *CHI* and *F3H* expressions are linked to higher accumulation of catechin. Similar to *PAL*, a massive up-regulation of *BIS3* expression was also observed. BIS3 is known to be the main BIS isoenzyme responsible for phytoalexin biosynthesis in apple shoots^7^. The expression of *BIS3* preceded the accumulation of noraucuparin, aucuparin and eriobofuran, suggesting involvement of *BIS3* in biphenyl-dibenzofuran biosynthesis. High *AOX* expression level in the VIE-treated ‘Florina’ cultures is probably associated with the enhanced biosynthesis of phenylpropanoids, as shown earlier in other plant species^61^. The elevated AOX expression is further known to be associated with the plant’s ability to facilitate metabolic re-programming to cope-up with stress conditions^62^.

The inhibitory effect of noraucuparin, aucuparin and eriobofuran on conidial germination of *V*. *inaequalis* provides further support for our assumption that biphenyl-dibenzofuran phytoalexins play crucial role in scab resistance in SR cultivar ‘Florina’. Here we observed that conidial germination is reduced by addition of these phytoalexins together (Supplementary fig. S2), suggesting a synergistic mode of actions of these phytoalexins. Together with the observation that these biphenyl and dibenzofuran phytoalexins were induced by VIE-treatment in ‘Florina’, this result supports the hypothesis that these specialized phytoalexin accumulation *in planta* have a role in prohibiting the scab infection and disease progression. However, the exact mode-of-actions of these phytoalexin need to be further investigated for better understanding of resistance mechanism in apple.

## Conclusions

This is the first report to use non targeted GC-MS metabolomics analyses of apple cell cultures treated with elicitor prepared from the scab fungus *V*. *inaequalis*. Our results showed that the developed metabolomics method has excellent sensitivity and specificity to analyze metabolites from apple cell cultures. Further, our results indicated that apple cell cultures could be used as a model system to understand apple-*Venturia* interactions in terms of metabolic re-programming. We were able to identify significant changes in the secondary metabolites in ‘Florina’ cell cultures, indicating metabolic re-programming of specific biosynthetic pathways after VIE-treatment. Based on these results we can conclude that biphenyl and dibenzofuran biosynthesis pathway plays a crucial role in providing scab resistance in the ‘Florina’ cell cultures. In the present study, we analyzed metabolic re-programming in the *V*. *inaequalis* elicitor treated apple cell culture system. However, future metabolomics analysis using scab-infected apple plants is required to get a complete picture of metabolic basis of scab resistance in apple. Our results would provide a strong basis for the future metabolomics analysis of apple plants to decipher marker metabolites associated with the scab resistance in apples.

## Methods

### Chemicals

Solvents used for extraction were of high-performance liquid chromatography grade. 2-phenylphenol (internal standard for gas chromatography) was purchased from SRL chemicals (India). N-methyl-N-(trimethylsilyl)-trifluoroacetamide (MSTFA), pyridine and all metabolite standards were obtained from Sigma.

### Plant material and elicitor-treatment

One scab resistant (SR) apple (*Malus domestica*) cultivar ‘Florina’ and one scab-susceptible (SS) cultivar ‘Vista Bella’ was used in this study to develop cell suspension culture. Cell suspension cultures were developed as described before^2^. Cell cultures were grown in liquid LS medium containing 2 µM 2,4-D and 1 µM NAA at 26 °C in the dark on an orbital shaker at 120 rpm. An elicitor prepared from apple scab fungus, *V*. *inaequalis* was used in this study. *V*. *inaequalis* strain (MTCC No.: 1109) was purchased from microbial type culture collection and gene bank (MTCC), Chandigarh, India. *V*. *inaequalis* elicitor (VIE) was prepared by homogenizing the fungal hyphae as described before^63^. Briefly, 10 g of ground fungal mycelium was added to 1 L of acidified water (pH 2.0). Water extract was then boiled for 1 h on a hot plate, cool down to room temperature and filter sterilized. After filtration, the pH of the fungal extract solution was adjusted to 5.0 and the final volume was adjusted to 1 L by adding distilled water. This solution was used as the VIE. Elicitor-treatment was performed by adding 2.5 mL of the VIE (~70 mg of fungal polysaccharide) to the 50 mL of seven-day-old cell apple suspension cultures of both ‘Florina’ and ‘Vista Bella’. Upon elicitor-treatment, cells were harvested at defined time points: 0, 6, 12, 24, 36, 48, and 72 hpe. In control treatment, similar volume of sterile distilled water was added in lieu of the VIE. All experiments were performed with at least three biological repeats.

### Extraction of polar metabolites

The VIE-treated apple cell cultures were harvested by vacuum filtration at defined time points (0–72 h) and kept in hot air oven at 60 °C for 4 h. Dried cell mass (2 g) was crushed in liquid nitrogen and fine powdered samples were used for metabolite extraction. The extraction of polar metabolites for GC-MS analyses was performed following the protocol described before^64^ with suitable modifications. An extraction mixture was prepared by adding methanol/water/chloroform in the ratio of 2.5:1:1 (v/v/v) and stored at −20 °C. Pre-cooled extraction mixture (1 mL) was then added to 200 mg powdered samples in a 1.5 mL micro centrifuge tube and vortexed vigorously at room temperature for 2 min. In order to identify extraction efficiency, 50 µL of 2-phenylphenol (from 2 mg.mL^−1^ methanol stock) was spiked in the extraction mixture as the internal standard (IS) and vortexed again for 1 min. The extracts were then centrifuged at 14000 g for 5 min. The resulting supernatant (0.8 mL) was transferred into a new 1.5 mL tube and then 0.4 mL of water was added to the supernatant, whole mixture was vortexed for 10 s and subsequently centrifuged at 14000 × g for 5 min. The polar upper phase (methanol/water) was transferred to a new micro-centrifuge tube and then dried out in a vacuum concentrator (Labconco, Centrivap; USA) at 20 °C for 2 h followed by 12 h freeze drying in a lyophilizer. Finally dried material was subjected to double derivatization for GC-MS analyses^65^. First, derivatization was performed by adding 40 μL of methoxyamine hydrochloride (stock solution: 20 mg.mL^−1^ in pyridine) to the dried sample and incubating the solutions at 37 °C for 2 h. Then second derivatization was performed by adding 80 μL of N-methyl-N-(trimethylsilyl)-trifluoroacetamide (MSTFA) at 37 °C for 30 min. Derivatization reaction prepared with empty tube served as the control.

### GC-MS analysis

GC-MS analysis was performed on Agilent 7890A gas chromatograph (Agilent technologies, CA, USA) coupled with an Agilent 5975C mass detector (Agilent technologies, CA, USA). Double-derivatized sample (1 μL) was injected into GC-MS by automatic sampler (7683B series, Agilent Technologies) with a split ratio of 1:5. DB-5 MS column (5% phenyl methyl polysiloxane: 30 m × 0.25 mm i.d. × 0.25 µm, Agilent technologies) was used for metabolite separation. The temperature program was as follows: Initial temperature of 80 °C for 1 min, followed by temperature increase to 220 °C at the ramp rate of 10 °C.min^−1^, followed by temperature increase to 310 °C at the ramp rate of 20 °C.min^−1^ and finally a 10 min hold at 320 °C. Total run time calculated was 39 min. Helium was used as carrier gas at a flow rate of 1 mL.min^−1^. The inlet temperature and interface temp was set 280 °C. The MS unit was tuned to its maximum sensitivity and the mass range for total ion current was m/z 80–700, and the detector voltage was set at 1700 V. Each sample was replicated three times. Scan was started after solvent delay of 7 min with scan frequency of 4 S^−1^ (2.0 HZ).

### Metabolite identification

Metabolites were identified by comparing the mass-to-charge ratios and abundance of each metabolite detected against a standard NIST-17 mass spectral library (National Institute of Standards and Technology), and our in-house mass spectral database that include several secondary metabolites, amino acids, organic acids, and sugar standards. Metabolite identity was reported only when the matching value of the mass spectra comparison was more than 70 percent, and an increase in the area of the corresponding peak was observed when spiking the sample with the corresponding pure standard. Each mass spectrum was carefully analyzed for co-elution detection. Co-elution was not detected in any of the identified peaks.

### Metabolite data pre-processing and statistical analysis

Raw GC-MS data files obtained from Agilent ChemStation^TM^ software were deconvoluted by Automated Mass Spectral Deconvolution and Identification System (AMDIS) using tools available with WsearchPro (www.wsearch.com.au). Metabolite data obtained were further converted into.csv (comma separated values) format before uploading in MetaboAnalyst 4.0 (http://www.metaboanalyst.ca). TIC values were normalized using internal standard. After that, pareto scaling (mean-centered and divided by the square root of standard deviation of each variable) was performed followed by normalization before statistical analyses. Significant differences in metabolite levels were calculated by one-way analysis of variance (ANOVA) using Statistical Package for the Social Sciences (SPSS) software followed by Tukey’s significant-difference test. Statistical significance level was set at *p* < 0.05. Principal component analyses (PCA) were performed by using interactive online tool of MetaboAnalyst 4.0. The output for PCA data consisted of score plots for visualizing the contrast between various time points of VIE-treated samples and loading plots to explain the cluster separation. A heatmap was created using interactive heatmap tool of MetaboAnalyst 4.0. A simplified metabolic pathway was manually constructed using information from the Kyoto Encyclopedia of Genes and Genomes (KEGG) database via pathway analysis in MetaboAnalyst 4.0.

### Quantitative Real-Time PCR

Total RNA was isolated from the VIE-treated ‘Florina’ cultures at defined time points (0–48 hpe) using RNeasy Plant Mini Kit from Qiagen (www.qiagen.com). Total RNA (1 µg) was reverse transcribed at 42 °C using Oligo (dT) primers and RevertAid H Minus reverse transcriptase (Fermentas; www.thermoscientificbio.com) to form cDNA. Quantitative RT-PCR was performed with the QuantStudio 3 Real-Time PCR System (Thermo Fisher Scientific) using Power Up^TM^ SYBR Green Master Mix (Thermo Fisher Scientific) following the manufacturer’s instruction. PCR program: 40 cycles at 90 °C for 15 sec followed by 55 °C for 1 min and final extension at 72° C for 1 min. Melt-curve analysis was performed to evaluate gene-specific amplification. Amplification and correlation efficiencies of each PCR were determined using six serial dilutions of cDNA from all samples. To derive relative quantification, the PCR efficiency was used to transform the cycle threshold values into raw data. Expression levels of *M*. *domestica phenylalanine ammonia-lyase (MdPAL)*, *cinnamate-4-hydroxylase* (*MdC4H*), *4-coumarate CoA ligase* (*Md4CL*), *chalcone flavanone isomerase* (*MdCHI*), *flavanone 3-hydroxylase* (*MdF3H*), *biphenyl synthase 3* (*MdBIS3*), and *alternative oxidase* (*MdAOX*) were evaluated using the gene-specific primers (Supplemental Table S3). All samples were normalized using apple *actin* gene. Scaling of expression level was performed in relation to the respective mRNA expression levels in the control (0 h) cells, which were set to 1. Three technical repeats were performed. Published mathematical model was used for the estimations of efficiency and gene expression levels^66^.

### Conidia germination inhibition assay

*V*. *inaequalis* mycelium was grown on potato dextrose agar plates covered by cellophane paper and conidia were harvested as described before^67^. Conidia were suspended in autoclaved water in a concentration of 1 × 10^5^ conidia.mL^−1^. Conidia germination inhibition assay was performed as described earlier^68^. An aliquot (40 µL in 50% methanol) comprised of aucuparin, noraucuparin and eriobofuran, 5 µM each, either individually or in combinations, was mixed with 10 µL of conidial suspension (1 × 10^5^ conidia.mL^−1^). After mixing well, 10 µL was immediately plated on 2% water-agar slides. In control slides conidia were incubated in presence of 50% methanol. After 24 h incubation at 20 °C the number of germinated conidia of both treated and control slides were counted under microscope.

The inhibition percentage of conidial germination was calculated using following the formula:

$${\rm{Inhibition}}( \% )=100-(\mathrm{C1}/\mathrm{C2})\times 100$$

C1 = Number of germinated conidia in treated slides.

C2 = Number of germinated conidia in control slides.

## Electronic supplementary material

Supplementary Data

Supplemetary Table S1

Supplementary Table S2

Supplementary Table S3

## Data Availability

All data generated and analyzed in this study are included in this manuscript either as main data or as supplementary information files.
