# Supplementary material for: Comparative metabolomics of scab-resistant and susceptible apple cell cultures in response to scab fungus elicitor treatment
Source: Sci Rep. 2018 Dec 14;8:17844. doi: 10.1038/s41598-018-36237-y (PMC6294756; doi:10.1038/s41598-018-36237-y)
Supplement: Supplementary file 1 — Supplementary Data [file 41598_2018_36237_MOESM1_ESM.pdf]

Comparative metabolomics of scab-resistant and susceptible apple cell cultures in response to scab fungus elicitor treatment

Amol Sarkate[1#], Shashank Sagar Saini[1#], Deepa Teotia[1], Mariam Gaid[2], Javid Iqbal Mir[3], Partha Roy[4], Pawan Kumar Agrawal[5*], Debabrata Sircar[1*]

Supplementary information

Supplemental Figure S1. GC-MS chromatograms (TICs) of VIE-treated (36 hpe) cell culture of apple cultivar 'Vista Bella' and 'Florina'.

Supplemental Figure S2. Effect of supplementation of induced cell culture metabolite noraucuparin (NA), aucuparin (A) and eriobofuran (E) on the conidial germination of scab fungus *V. inaequalis*. The ANOVA was performed to assess the statistical significance of differences between treatment ($p < 0.001$). Results are means ± SD ($n = 3$).

Supplemental Table S1. Total number of metabolites detected by GC-MS analyses from the VIE-treated cell suspension culture of scab resistant (SR) apple cultivar 'Florina' and scab susceptible (SS) cultivar 'Vista Bella'. 'ND' denotes metabolites detected only in 'Florina' and not in 'Vista Bella'

Supplemental Table S2. Total number of metabolites detected from VIE-treated cell cultures of 'Florina'. Differential metabolite accumulation was analyzed with Bonferronio corrections ($p < 0.05$). Differential accumulations were expressed as significant (S; $p < 0.05$) or non significant (NS; $p > 0.05$).

Supplemental Table S3. Primers used in gene expression analyses by qRT-PCR

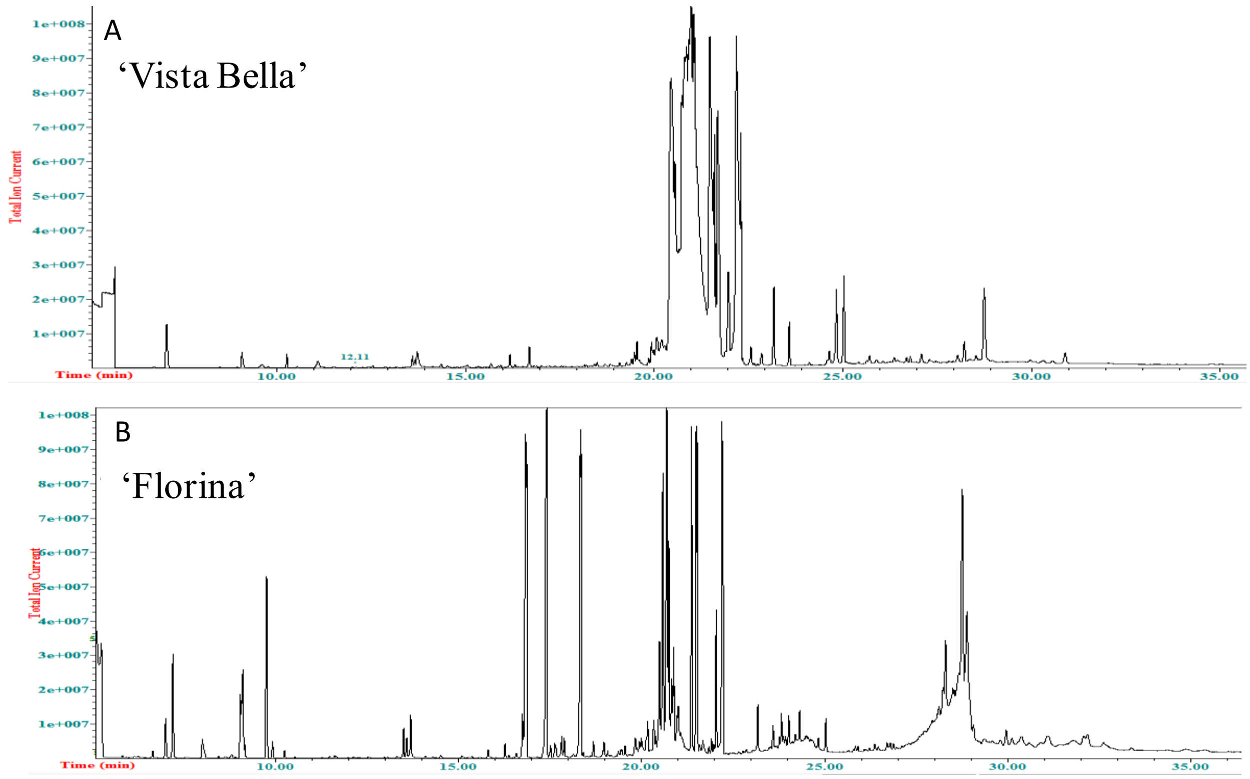

Supplemental Figure S1. GC-MS chromatograms (TICs) of VIE-treated (36 hpe) cell culture of apple cultivar 'Vista Bella' and 'Florina'.

.

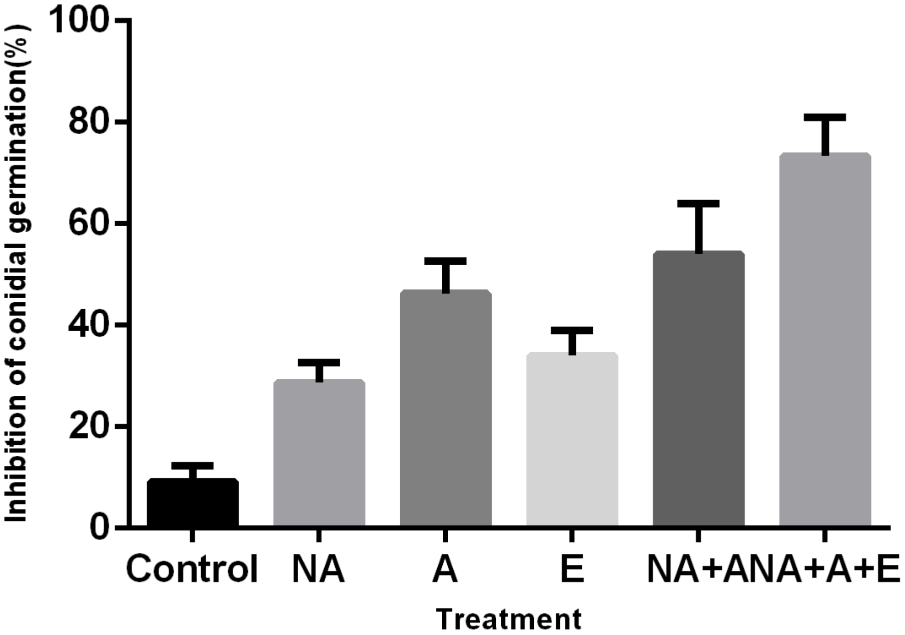

Supplemental Figure S2. Effect of supplementation of induced cell culture metabolite noraucuparin (NA), aucuparin (A) and eriobofuran (E) on the conidial germination of scab fungus *V. inaequalis*. The ANOVA was performed to assess the statistical significance of differences between treatment ($p < 0.001$). Results are means ± SD ($n = 3$).
